# Instable Microdeformation and Strain Recovery in Amorphous LiPON Thin Layer

**DOI:** 10.1021/acsomega.4c07378

**Published:** 2024-12-17

**Authors:** Dávid Ugi, Alexandra Musza, István Groma, Jens Glenneberg, Julian Schwenzel, Péter Dusán Ispánovity, Robert Kun

**Affiliations:** † HUN-REN Research Centre for Natural Sciences, Institute of Materials and Environmental Chemistry, Magyar Tudósok Körútja 2, 1117 Budapest, Hungary; ‡ Department of Materials Physics, ELTE Eötvös Loránd University, Pázmány Péter sétány 1/a, 1117 Budapest, Hungary; § Department of Industrial Materials Technology, Production Division, Bay Zoltán Nonprofit Ltd. for Applied Research, Kondorfa utca 1, 1116 Budapest, Hungary; ∥ Fraunhofer Institute for Manufacturing Technology and Advanced Materials IFAM, Wiener straße 12., 28359 Bremen, Germany; ⊥ Department of Chemical and Environmental Process Engineering, Faculty of Chemical Technology and Biotechnology, Budapest University of Technology and Economics, Műegyetem rkp. 3, 1111 Budapest, Hungary

## Abstract

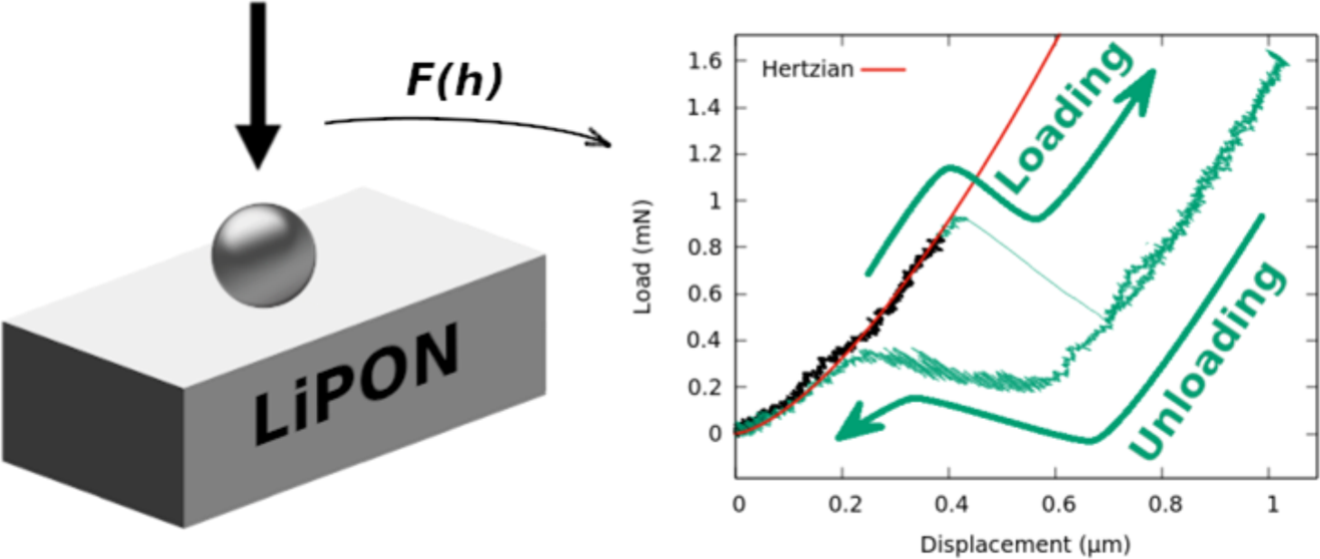

Lithium phosphorus oxynitride (LiPON) is a crucial electrolyte for all-solid-state thin-film batteries due to its sufficient ionic conductivity. Understanding the mechanical behavior of LiPON films is crucial for further technological development. Previous studies noted unexpected ductility and strain recovery in amorphous LiPON during sharp-ended tip indentations revealing pile-up formation and densification as the main deformation mechanisms. Our work presents nanoindentation experiments including spherical tips, revealing a novel mechanical behavior of a sudden deformation event followed by slower but complete strain recovery during unloading. This unique deformation phenomenon is likely linked to the material’s special structure, featuring isolated phosphate tetrahedra P(O,N)_4_ embedded in an amorphous Li matrix with occasional N bridge bonds between tetrahedra. In this study, the authors report on a range of nanoindentation experiments, examining how instability depends on strain rate and the indenter’s tip geometry. It is found that instability occurs only within a specific range of deformation velocities and strongly depends on the indenter’s tip sharpness. Assuming the mobility and the capability of the cooperative movement of the tetrahedra, the measured novel deformation method, and other, deformation-attached properties of the LiPON can be explained.

## Introduction

Over the past decades, the importance of the battery technology has increased significantly as there is an ever growing need for portability and high storage capacity in a compact size to store the chemical energy and to convert it into electrical energy. There are many battery types, for example heavy-metal - acid, but arguably lithium ion (Li-ion) batteries have by far the most applications in portable electronics and electric vehicles. Batteries made of lithium are much lighter than nickel-based ones and are also more durable since no crystals form in the battery at all. The mainstream type of Li-ion batteries contain liquid electrolytes, but exhibit many drawbacks, such as limited voltage, poor mechanical strength and flammability.^1−3^ The solid-state batteries (SSBs), on the other hand, are potentially much safer.^4,5^ SSBs can utilize metallic lithium for the anode, making it possible to achieve high energy density, and employ a separator that ideally allows only lithium ions to pass through.^6^ Since its discovery in the early 1990s,^7^ LiPON (lithium phosphorus oxynitride, Li_*x*_PO_*y*_N_*z*_) has been one of the most popular solid-state electrolytes used for planar lithium ion microbatteries. The success of LiPON thin-film electrolytes can be attributed to their excellent properties such as small thickness, good ion conductivity at room temperatures, high electronic resistivity, and unmatched long-term durability in terms of cycling performance and elastic energy storage capability..^8−11^

### Chemical Properties

Bates et al. showed that the ionic conductivity of LiPONs increases significantly with increasing atomic percentage of N.^7^ There are several ideas about how nitridation affects the structure, which are based on the fact that increasing number of N atoms promotes cross-linking by the formation of double (N_d_) and triple (N_t_) coordinated N bridges between P atoms.^12,13^ According to one theory, Li-ion mobility is caused by the mixed anion effects created by nitriding.^14,15^ From an electrostatic point of view, the different levels of covalency of P–N bonds concerning P–O, would affect the interaction with Li^+^ and cause different ion conductivity.^16^ Lacivita et al. investigated the Li^+^ mobility in the amorphous LiPON electrolyte using ab initio molecular dynamics methods. They found that the mobility is strongly influenced by the chemistry and connectivity of phosphate polyanions near Li^+^^17^ Complementing molecular dynamics with infrared spectroscopic experiments, it was determined that N forms both bridges between two phosphate units and nonbridging apical N (N_a_).^18^ According to the study by Yu et al., in addition to the fact that nitrogen is built into the structure of the deposited film and increases the electrical conductivity, it is electrochemically and mechanically stable, thus LiPON can also form a barrier against dendrites growing out of the Li anode.^8^

### Mechanical Properties

Based on previous studies, it can be said that the mechanical stress causes roughening of the anode, which creates metallic protrusions that lead to the formation of dendrites.^19^ Li forms dendrites during repeated cycling that may lead to short circuits, thermal runaway, and explosion hazards.^20^ However, since this phenomenon has been in the focus of attention, investigations have also taken new directions and these studies have paved the way toward safer batteries. According to some studies, mechanical behaviors of the involved constituents play a critical role in the formation and suppression of Li dendrites and the corresponding interfacial stability.^19,21^ Jana and Garcia investigated dendrite morphology and concluded that growth is a direct product of the competition between the rate of Li deposition and the plastic deformation of Li under pressure,^22^ that is, the morphology of lithium is strongly dependent on the charge rate and feature size. There is a theory that dendrite formation can be prevented if the shear modulus of the electrolyte is about twice that of the metal anode and this value may be sufficiently high to mechanically suppress dendrite formation at the lithium/LiPON interface in thin-film batteries.

In the study of Glenneberg et al. the morphological and electrochemical changes of LiPON under different external stress situations were investigated in a unique way. They employed bending experiments and observed that decreasing bending radii lead to a decrease in the LiPON resistance and also to reduced activation energies for the lithium migration as a result of the internal stress within the electrolyte layers, due to bending.^23^

Kalnaus et al. investigated the resistance to cracking (fracture toughness) of LiPON by nanoindentation.^24^ During the nanoindentation it was observed that the localized stress supporting the indenter tip can be relieved by three major mechanisms: densification (which appeared recoverable at room temperature), constant volume (isochoric) shear flow, and formation of new surfaces via fracture^25^ and observed ductility and the ability to strain recovery^26^ in this material (it was not possible to induce cracks).

In this paper, authors focus on the micromechanical properties of LiPON thin films, since these parameters could crucially affect the electrochemical performance of SSBs. It was aimed at finding a possible explanation for a less-known strain recovery capability of LiPON, which could play a main role in the ion conductivity of the solid-state electrolyte.

## Materials and Methods

### Sample Preparation

LiPON thin films are commonly deposited using reactive sputtering of a Li_3_PO_4_ target in an N_2_ atmosphere^27,28^ or physical vapor deposition (PVD), such as sputtering.^29^ Our layers were prepared according to the synthesis protocol described in our previously published paper.^23^ These layers were sputtered via RF-Sputtering using a 4″ Li_3_PO_4_ target (Plasmaterials Inc.). In order to deposit the LiPON onto smooth synthetic sapphire substrate (due to its chemical and mechanical resistivity) an RF power of 120 W was used, while having a sputter pressure of 2 × 10^–1^ Pa and a gas flow of 100 sccm (Standard cubic centimeters per minute) dry nitrogen. The Li/P ratio widely utilized in the literature and known to influence the structure-was indirectly controlled, and stemming from the target’s composition and PVD process parameters.

Sputtering for a total of 5 h led to a LiPON thickness of around 1 μm, which was verified by FIB-SEM studies. Based on XPS-studies a composition of Li_2.13_PO_2.47_N_0.67_ was determined for the sputtered LiPON,^23^ which is in perfect agreement with literature values.^30,31^ According to the apparatus supplier (MBraun), the used 4-in. target in our setup (fixed substrate-to-target distance, substrate carrier rotation at 30 rpm) allows for homogeneous lateral distribution (both in-plane and thickness). The obtained layer exhibited exceptional homogeneity, with no detectable variations in thickness or chemical composition.

### Nanoindentation

The in situ indentations were carried out at room temperature inside a Mbraun-MB200B glovebox with Ar atmosphere and oxygen and water content less than 0.1 ppm. A custom-made nanoindenter was used without any load or strain feedback loop integrated. Instead of the traditional controlling modes, a constant platen velocity was applied during the tests which characterized the average strain rate, as in the case of previous studies.^32,33^ The application of this natural-like controlling allows precise investigation of the stress-releasing and -accommodating mechanisms. During deformation, one end of a spring (having a spring constant of *k* = 1.72 mN/μm) was attached to the indenter tip while the other end was moved at a constant (platen) velocity *v*_p_. The controlling of the spring involves both loading and unloading phases, each executed at identical platen velocities but in opposite directions. Between these phases a holding phase was carried out, lasting half the duration of the loading phase. A total of 122 nanoindentation experiments were conducted (Table 1.) employing varied platen velocities ranging from 1 to 40 nm/s. Three distinct indenter tips were utilized, including two spherical ones with radii of 2 (named “Spherical 2’’) and 10 μm (named ′′Spherical 10’’), as well as a sharp-type Berkovich indenter.

**Table 1:** Number of Executed Indentation Experiments at the Given Platen Velocity, Depending on the Applied Tip’s Geometry^a^

	Spherical 10					Spherical 2					
υ_p_ (nm/s)	ratio	*A* (nJ)	*F*_*y*_ (mN)	*h*_*y*_ (nm)	slope	ratio	A (nJ)	*F*_*y*_ (mN)	*h*_*y*_ (nm)	Slope	Berkovich ratio
1											0/2
3						0/2					0/2
5	0/2					3/9	0.128	0.89	165	0.07	0/2
7.5						3/9	0.049	0.72	195	–0.96	
10	0/3					3/9	0.070	0.98	348	–1.57	0/2
12.5						0/9					
15	0/4					0/9					
17.5	2/9	0.183	2.59	300	1.23	0/7					
20	2/9	0.248	2.61	385	0.06	0/2					0/2
22.5	0/9										
25	2/9	0.228	3.67	440	–0.32						
30	0/9										
40											0/2

a Deformation instability was found where *Ratio* is not zero. There, the area A of the hysteresis, the initiation force *F_y_* of the instability, the related depth at instability *h_y_*, and their Slope (refers to the velocity of the loading part of the deformation event pair) are presented.

### Mechanical Properties

A spherical tip with radius *R* is indented to a depth *h* by an applied force *F*. Assuming that the material is elastically isotropic and behaves according to the Hertzian theory,^34^ the indenter has a contact radius *a* with the material, given by
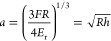
1where *E*_r_ is the reduced Young’s modulus given by
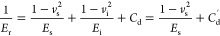
2

Here, *E*_i_ and *E*_s_ are the moduli of the indenter’s tip and specimen, respectively; similarly, *V*_i_ and *V*_s_ are the Poisson ratios for the tip and the specimen,^34,35^ finally, the additive term *C*_d_ is due to the additional elasticity originating from the frame and the natural imperfection of the sample supporting system of the device. The Load–Displacement *F*(*h*) function can be given in the early loading regime as
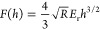
3

In the case of Berkovich indentation, the conventional approach for elasticity calculation is outlined in.^36^ However, in this paper, in order to match the results of spherical indentations, an alternative method was employed to describe the elastic regime. In total 110 indentation experiments were carried out with spherical tips and *E*_s_ and *V*_s_ were obtained from literature data: the modulus was found to be *E*_s_ = 73 GPa on average, and the Poisson’s ratio of LiPON was found to be *V*_s_ = 0.25.^26,36,37^*C*_d_^′^ was then considered as a fitting parameter, and could be calculated from (3). In the case of a 10 μm spherical indenter, the *C*_d_^′^ parameter was determined as *C*_d_^′^ (Sph10) = 0.196 ± 0.03 GPa^–1^, while, for the 2 μm tip *C*_d_^′^ (Sph2) = 0.088 ± 0.01 GPa^–1^ was obtained.

To calculate the estimated radii and *C*_d_^′^ parameter (elastic contribution of the device) of the Berkovich indentations, it was assumed that the *C*_d_^′^ parameter is dependent on the sharpness of the indenter’s tip, where the use of a less sharp tip contributes more to the *E*_r_ value via its less capability of penetration. We were using the *R*^1/2^*E*_r_ product of (3) as the fitting parameter. Moreover, based on the two types of spherical indentations it could be seen that *C*_d_^′^ changes with the same multiplicator as *R*^1/2^.

Thus, in the case of the Berkovich tip, it was assumed that *R*_Berk_^1/2^, and *C*_dBerk_^′^ changing with the same m_2&berk_ compared to the parameters of the spherical tip with radii of 2 μm, and *R*_berk_^1/2^*E*_r_(berk) = 12.34 μm^1/2^ GPa comes from the fitting (Figure S4a). It resulted, that *R*_berk_ = 0.46 μm; *C*_dberk_^′^ = 0.042 GPa^–1^; *m*_2&berk_ = 0.48.

## Experimental Results

**Figure 1:**
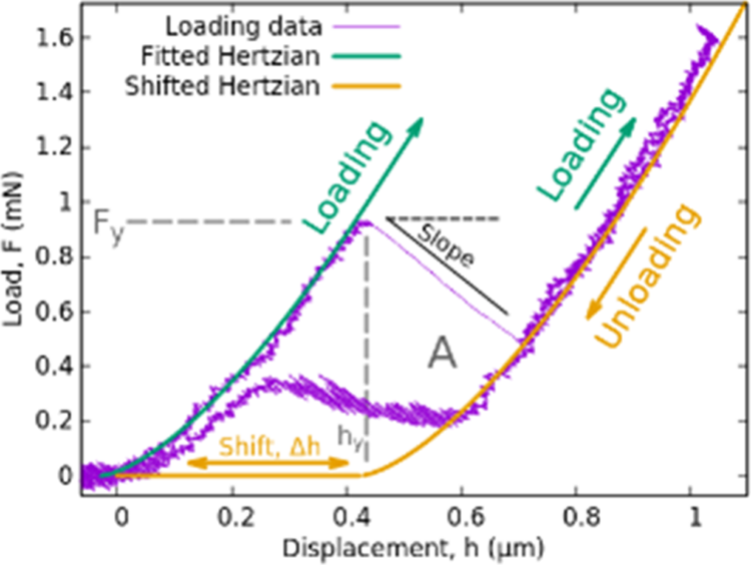
One of the typical indentation curves containing deformation instability and the calculated parameters presented in Table 1.

In this study, the reported novel deformation phenomenon is described as follows. Figure 1 plots a particular load–displacement curve with purple color. The displacement was calculated as the position of the indenter’s tip relative to the initial tip–sample touch. This spectacular deformation phenomenon initially starts with a fully elastic regime. In that early stage of the loading a Hertzian curve of eq 3 can be fitted perfectly as indicated by the green curve with an arrow in Figure 1. This phase is followed by a sudden deformation event characterized by a notable increase in the displacement from 0.4 to 0.7 μm. Following this event, deformation continues to remain elastic again, and follows a shifted Hertzian curve. However, during unloading, a strain recovery is observed at a smaller force than that of the initial strain burst, i.e, a the curve exhibits hysteresis. Remarkably, no residual deformation is detectable after the indentation, suggesting the absence of any conventional “irreversible’’ plastic deformation. The duration and magnitude of this type of deformation event can vary significantly, as it is explained below.

Table 1 summarizes the conducted experiments and some of the calculated parameters, separated by the platen velocities and the type of the tips. The columns named “Ratio’’ show the number of executed experiments in a given platen velocities, and the number of indentations where the deformation instability detailed above was unambiguously observed. In a given row, the values of *A*, *F*_*y*_, *h*_*y*_, and *Slope* parameters represent the averages. These values (explained in Figure 1) characterize the stored energy during the cycle (*A* as the area of the hysteresis), the force and displacement at the onset of the plastic event *F*_*y*_, *h*_*y*_ and the *Slope* depends on the rate of the event.

**Figure 2:**
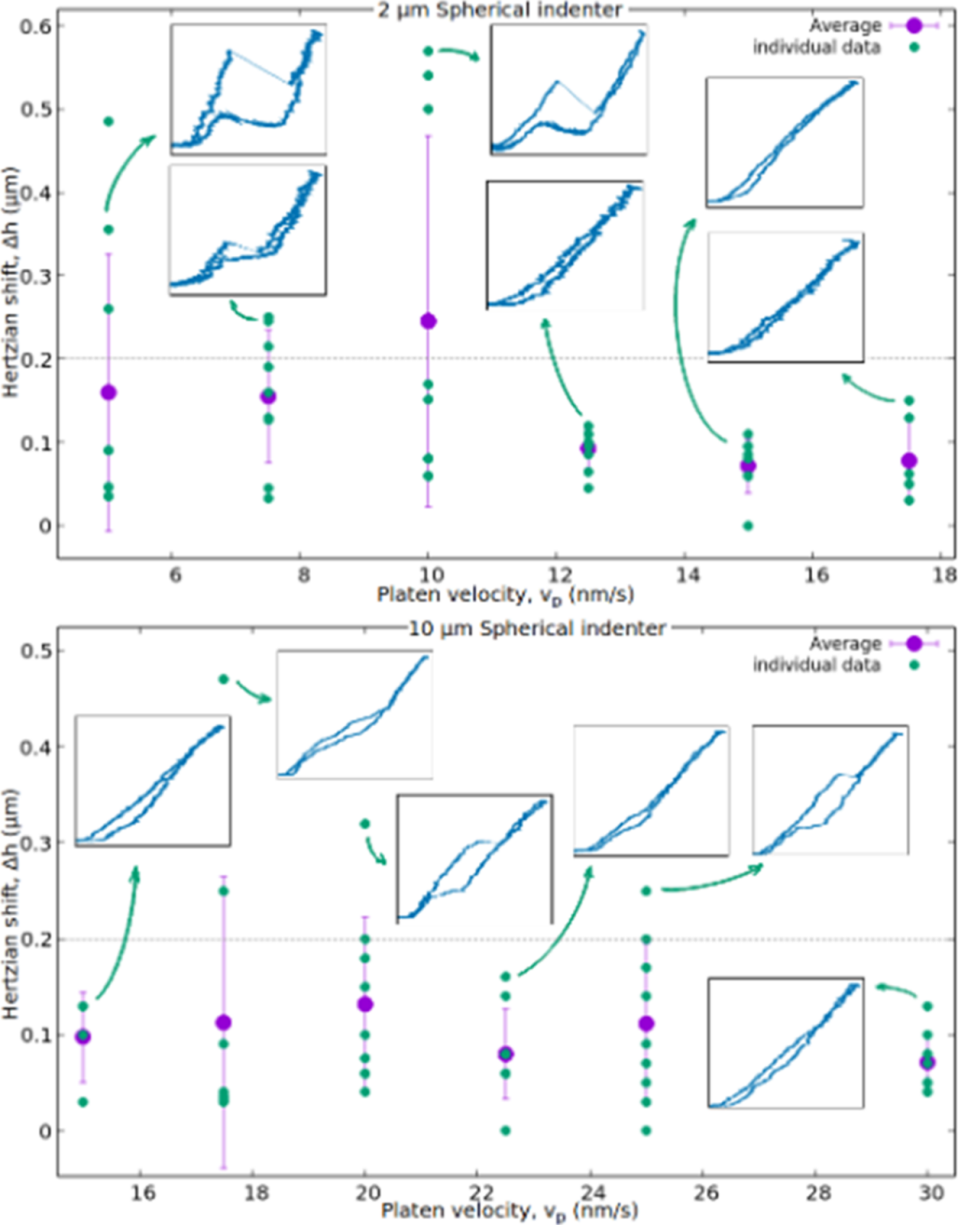
Parameters of the displacements Δ*h* for individually fitted Hertzian curves in the case of single indentation measurements are shown in green, while their per-velocity averages are depicted in purple. The cases of the spherical indentation with 2 μm radii at the top (where instability occurred at lower velocities), and 10 μm at the bottom. Insets provide examples of force–displacement curves for individual experiments, marked with green arrows.

The most important velocity dependence of our data is the presence of deformation instability. To define the occurrence of instability, we based our analysis on the fits shown in Figure 1. The peculiar nature of these instabilities is that, following the sudden displacement burst, it is followed by the Hertzian curve corresponding to the initial elastic region, shifted along the displacement with Δ*h*. After fitting the initial elastic region, the Hertzian curve associated with the unloading part, except for the parameter Δ*h*, can generally be undoubtedly identified. As a criterion for the existence of instability, we chose an artificially selected threshold value for Δ*h*. If the Δ*h* value for the two fitted curves on the load–displacement graph exceeded 200 nm, we considered it an indication of instability. Based on Table 1. these instabilities occur at high platen velocities in the case of the spherical tip with 10 μm radii, while at slower velocities for 2 μm spherical indentation (Figure 2), and are not present at all for the sharp Berkovich tip. On the other hand, all the investigated parameters show no significant dependency on the platen velocity.

**Figure 3:**
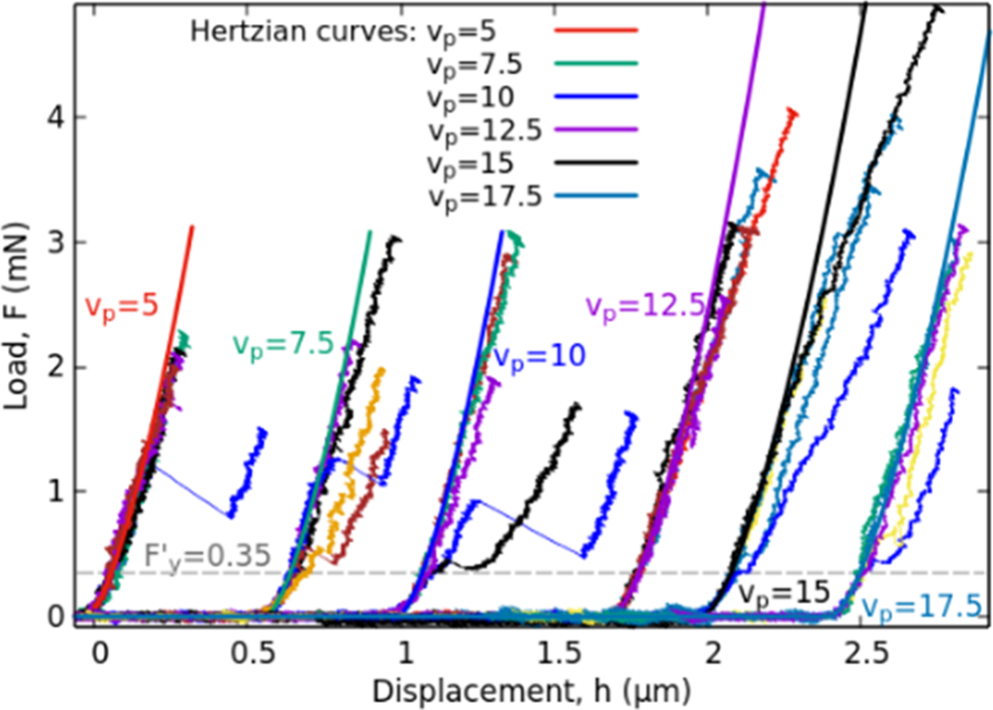
Loading parts of five–five representative load–displacement curves for different platen velocities (shifted along the *h* axes sorted by *v*_p_) for the spherical tip of 2 μm radius.

On the Figure 3. Thirty more representative loading parts of the indentations curves given by the 2 μm radii spherical indentation (organized by the platen velocities) were selected regarding visualization. These curves show that there are instabilities with different yielding points, rates, and strain burst sizes. (Also, more experimental data is available in the Supporting Information: Figures S1, S4a), moreover the whole loading–unloading parts in Figures S2–S4.

Numerous parameters can be associated with this reversible instability for characterization, allowing the derivation of some fundamental conclusions. The global yielding of the indentations, represented by the gray dashed horizontal lines in Figures 3, S1, S4a, and decreases with the increasing tip sharpness with the registered values of 0.75, 0.35, and 0.25 mN. Furthermore, in the case of spherical tips, the stored energy (proportional to A values) during the reversible deformation cycle decreases for sharper tips. Additionally, the duration of these events (inversely proportional to the *Slope*) also decreases for sharper tips.

## Discussion

In our investigation, we explored the mechanical properties of the solid-state electrolyte LiPON. Given that both elasticity and plasticity play pivotal roles in determining the durability of LiPON-based batteries,^21,22,38^ our research involved nanoindentation experiments employing diverse tip shapes and strain rates. Utilizing a specially designed nanoindenter,^33^ our controlling method differed from traditional methods that typically use force or strain control. This departure from conventions allowed us to unveil a novel deformation event linked to the intricate structure of the examined material. The strain recovery, an integral aspect of these complex deformation properties, has been previously documented in the literature.^24^

Previous studies have highlighted the exceptional elastic energy storage capacity of LiPON,^23^ emphasizing its resistance to crack formation and identifying potential deformation mechanisms such as hydrostatic densification and isochoric shear when surface pop-in events occur.^24^ Guided by our results (see Figures 1), we propose an alternative deformation mechanism to explain the reversible instability.

Assuming that the P(O,N)_4_ tetrahedra exhibit mobility within the amorphous Li matrix, akin to internal friction in a viscous medium, the local accommodation of these tetrahedra enables volume reduction (local deformation). This phenomenon arises from the higher density of the tetrahedra compared to the pure amorphous Li matrix. Moreover, the reversible instability observed in our study can be elucidated by considering the frictional mobility of these tetrahedra as well. Since with the decaying of the external stresses, the tetrahedras able to earn the initial homogeneous distribution.

The initiation of tetrahedral motion must occur at a certain force value F_*y*_. Once initiated, the avalanche-like cascade movements occur, representing a necessary condition for measurable deformation (and these stochastic properties generally accompany physical instabilities). This cascade effect propagates among neighboring tetrahedra, whereby the disappearance of a tetrahedron from its position creates a temporary vacancy, resulting in a higher density gradient in its proximity. This density gradient may provide the driving force to overcome the initial frictional forces, contributing to the cooperative tetrahedra movement.

Assuming the mobility of P(O,N)_4_ tetrahedra, during deformation even the chemical properties can change. According to simulations,^17^ if the Li/P ratio decreases, the tetrahedra can connect (increasing the number of the N_d_ bonds) and affect the Li + conductivity. If so, the deformation induced local P(O,N)_4_ accommodation can also affect the Li + diffusion (via the increased N_d_ bonds), which can explain the observation of Glenneberg et al.^23^ They observed, with increasing bending deformation, a decrease in the LiPON resistance and reduced activation energies for the lithium migration.

The cyclic properties of this deformation mechanism can be interpreted by Table 1. In the case of the less sharp Spherical 10 indentations, the stored energy during a cycle (proportional to *A*) is higher, which may result in the bigger activated volume via deeper h_*y*_ values. This stress-affected volume under the tip has lower inhomogeneity compared to the Spherical 2 tip, which may prevent the cooperativity of the tetrahedra. This could have caused the longer duration of the events and the positive values of the *Slope* parameters (slow deformation rate during events), which could indicate the tendency of cooperativity.

The reduced cooperativity of the tetrahedra in the case of sharp (or sharper) tip geometries could be attributed to the bigger inhomogeneity of the induced stress field under the tip. This implies that the volume activated by a less homogeneous stress field contains smaller regions with mechanical stresses exceeding the threshold needed to initiate the movement of the tetrahedra. This assumption can also explain the lack of instability in the Berkovich indentation, even if the tip possesses a nonperfect geometry.

## Conclusions

Understanding the mechanical behavior of LiPON films is crucial for further technological development, not only because of the durability of batteries, but also because the ion conductivity also depends on the deformation state of the LiPON.

In this study, authors reported the mobility of the P(O,N)_4_ tetrahedra within the amorphous Li matrix, akin to friction in a viscous medium. This capability can not only explain the reported experiments (unstable and sudden deformation event followed by most of the time total strain recovery), but also previously described phenomena such as enormous elastic energy storage capability, resistance to fracture, and deformation-dependent electrochemical properties.

Generally, a strain rate-dependent instability can be explained by a cooperative phenomenon, as demonstrated by previous studies.^33,39^ These cooperations exist between tetrahedra, exposed to a decent stress field. Since this field depends on the tip geometry, it can be inferred that the sharper the tip, the instability occurs with a lower probability. The varying level of cooperativity among tetrahedra can elucidate the absence of instability in the case of sharp indentation. Additionally, this novel deformation mechanism was not previously reported in the literature, as this study employed spherical-headed indenting controlled by different methods to unveil the unstable deformation.

## Supporting Information Available

The measurement curves of the numerous indentation experiments, from which the values in Table 1 and Figure 2 are derived, are provided comprehensively in the Supporting Information (see Figures S2, S3, and S4b,c). The complete set of measurement curves classified by the indenter tips corresponding to Figure 3 is presented in Figures S1 and S4a of the Supporting Information (PDF)
