# Supplementary material for: Instable Microdeformation and Strain Recovery in Amorphous LiPON Thin Layer
Source: ACS Omega. 2024 Dec 17;9(52):51221–7. doi: 10.1021/acsomega.4c07378 (PMC11696408; doi:10.1021/acsomega.4c07378)
Supplement: Supplementary file 1 — ao4c07378_si_001.pdf [file ao4c07378_si_001.pdf]

SUPPORTING INFORMATION

Instable microdeformation and strain recovery in amorphous LiPON thin layer

Dávid Ugi [1,2], Alexandra Musza [2,3], István Groma [2], Jens Glenneberg [4], Julian Schwenzel [4], Péter Dusán Ispánovity [2] and Robert Kun [1,5,]*

[1] HUN-REN Research Centre for Natural Sciences, Institute of Materials and Environmental Chemistry, Magyar Tudósok Körútja 2, 1117 Budapest, Hungary

[2] ELTE Eötvös Loránd University, Department of Materials Physics, Pázmány Péter sétány 1/a, 1117 Budapest, Hungary

[3] Department of Industrial Materials Technology, Production Division, Bay Zoltán Nonprofit Ltd. for Applied Research, Kondorfa utca 1, 1116 Budapest, Hungary

[4] Fraunhofer Institute for Manufacturing Technology and Advanced Materials IFAM, Wiener straße 12., 28359 Bremen, Germany

[5] Department of Chemical and Environmental Process Engineering, Faculty of Chemical Technology and Biotechnology, Budapest University of Technology and Economics, Műegyetem rkp. 3, 1111 Budapest, Hungary

[*]Corresponding author: Robert Kun
HUN-REN Research Centre for Natural Sciences, Institute of Materials and Environmental Chemistry, Magyar tudósok körútja 2., 1117 Budapest, Hungary
Tel: +36 1 382 6579
E-mail: kun.robert@ttk.hu

KEYWORDS: Solid state batteries; Li-ion batteries; In situ nanoindentation; Deformation instability; Strain recovery

1. Spherical-10 indentation

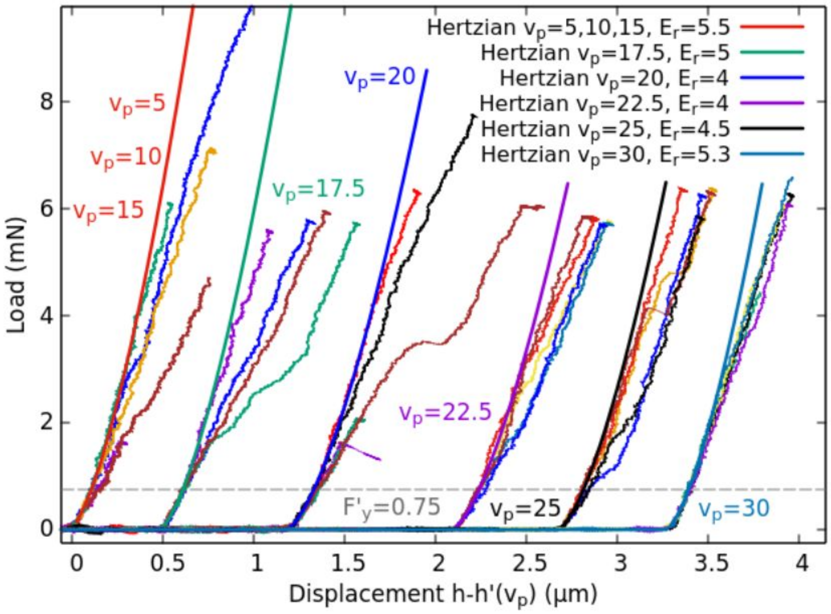

Figure S1. Loading parts of five-five representative load-displacement curves on different velocities, given by the spherical tip with 10 μm radii.

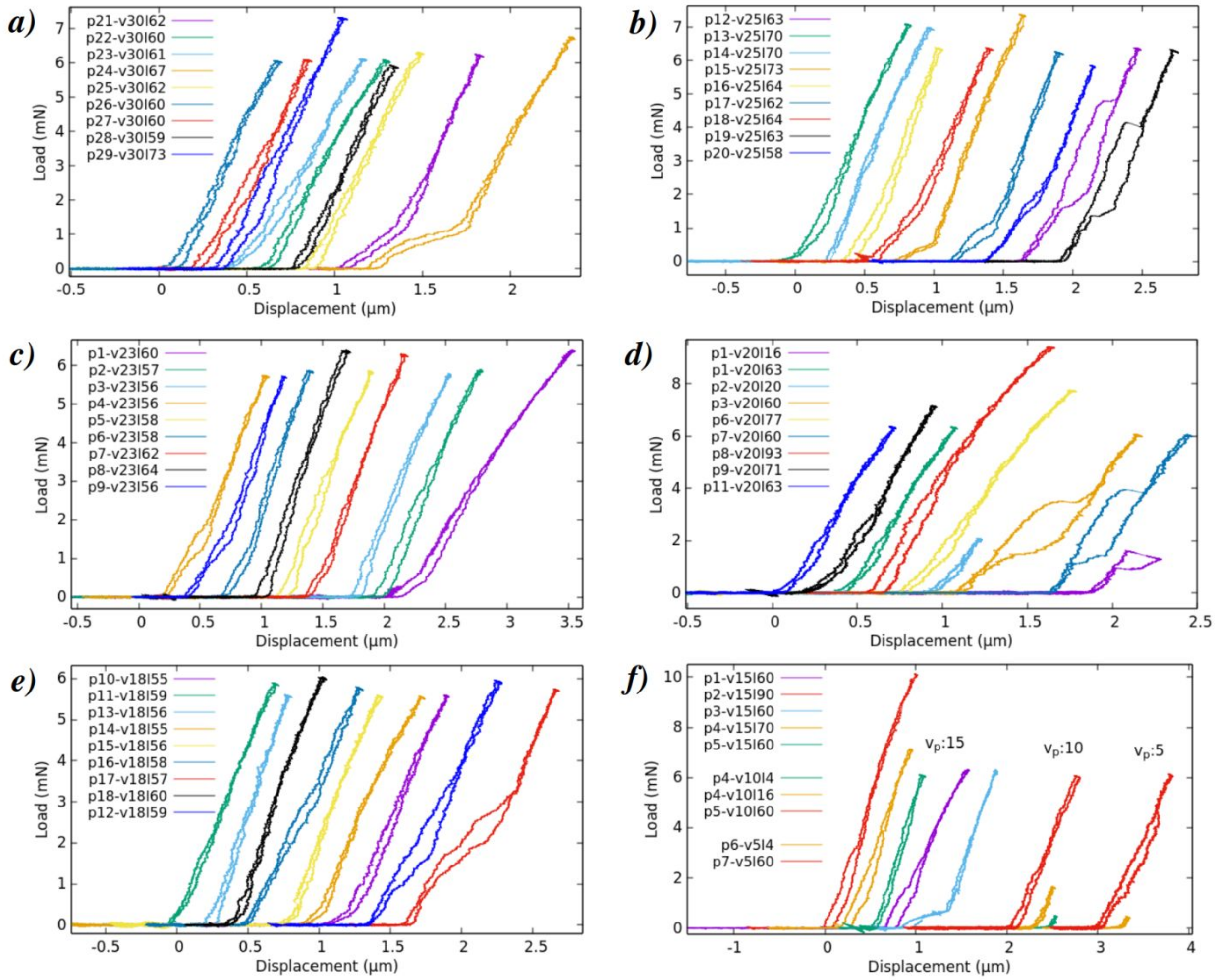

Figure S2. The Load-Penetration curves of indentations applied with the spherical tip with radii of 10 μm (The curves were shifted along the x-axis for better visualization). ***a)*** Applied platen velocity of 30 nm/s; ***b)*** Applied platen velocity of 25 nm/s; ***c)*** Applied platen velocity of 22.5 nm/s; ***d)*** Applied platen velocity of 20 nm/s; ***e)*** Applied platen velocity of 17.5 nm/s; ***f)*** Five curves on the left with platen velocity of 15 nm/s, three on the middle with 10 nm/s and two on the right with 5 nm/s.

2. Spherical-2 indentation

The curvature in high stresses is different, since at different velocities far spots were indented (not only the 20 μm step) which could possess different elastic contributions from the device.

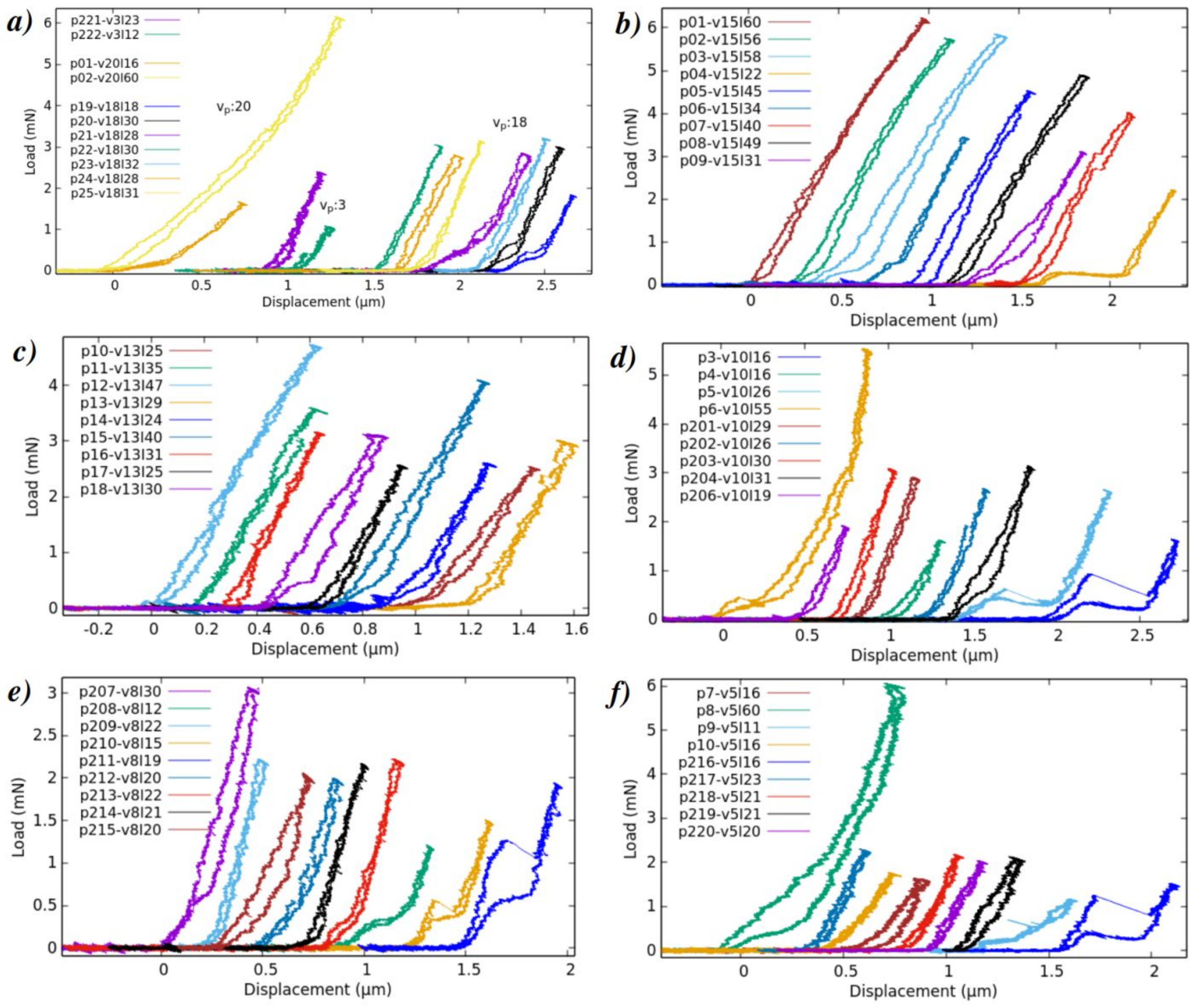

Figure S3. The Load-Penetration curves of indentations applied with the spherical tip with radii of 2 μm. (The curves were shifted along the x-axis for better visualization.) ***a)*** Two curves on the left with platen velocity of 20 nm/s, two on the middle with 3 nm/s and seven on the right with 17.5 nm/s. ***b)*** Applied platen velocity of 15 nm/s; ***c)*** Applied platen velocity of 12.5 nm/s; ***d)*** Applied platen velocity of 10 nm/s; ***e)*** Applied platen velocity of 7.5 nm/s; ***f)*** Applied platen velocity of 5 nm/s.

3. Berkovich indentation

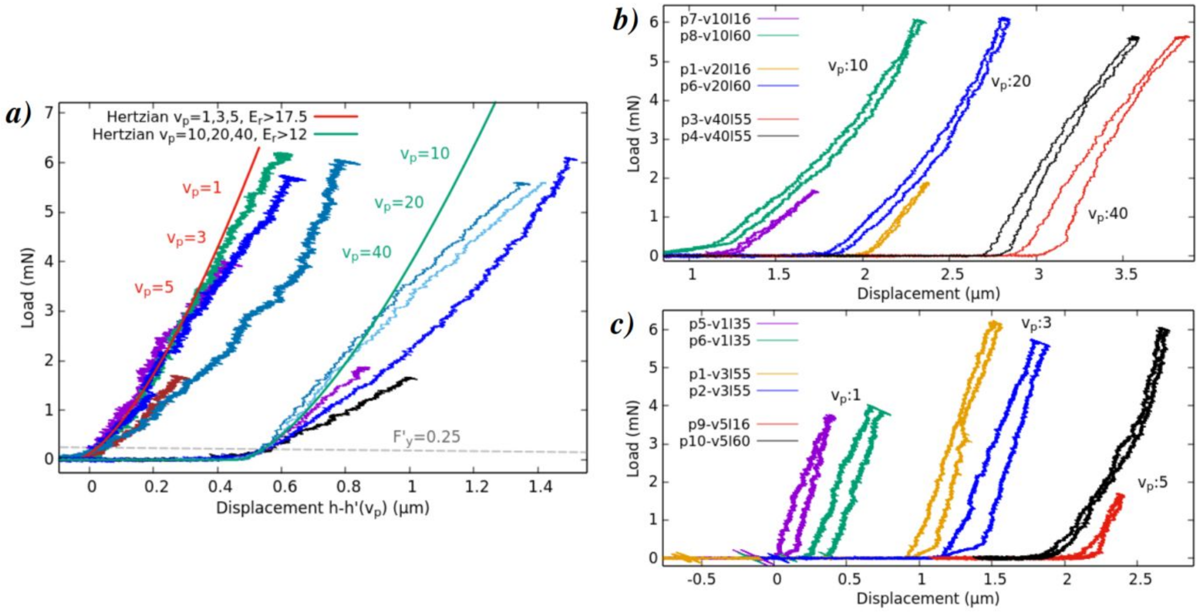

Figure S4. Loading data of indentations applied with the berkovich tip (The curves were shifted along the x-axis for better visualization). ***a)*** Loading parts of five-five representative load-displacement curves on different velocities. ***b)*** Load penetration curves: Two curves on the left with platen velocity of 10 nm/s, two on the middle with 20 nm/s and two on the right with 40 nm/s. ***c)*** Two curves on the left with platen velocity of 1 nm/s, two on the middle with 3 nm/s and two on the right with 5 nm/s.
